# Mechanisms of tumor-promoting activities of nicotine in lung cancer: synergistic effects of cell membrane and mitochondrial nicotinic acetylcholine receptors

**DOI:** 10.1186/s12885-015-1158-4

**Published:** 2015-03-19

**Authors:** Alex I Chernyavsky, Igor B Shchepotin, Valentin Galitovkiy, Sergei A Grando

**Affiliations:** 1 Department of Dermatology, University of California, 134 Sprague Hall, Irvine, CA 92697 USA; 2 National Cancer Institute, Kiev, Ukraine; 3 Department of Biological Chemistry, University of California, 134 Sprague Hall, Irvine, CA 92697 USA; 4 Cancer Center and Research Institute, University of California, 134 Sprague Hall, Irvine, CA 92697 USA

**Keywords:** Bronchial epithelial cells, Lung cancer cells, Nicotinic acetylcholine receptors, Proliferation, Growth factors, Intrinsic apoptosis, Mitochondria

## Abstract

**Background:**

One of the major controversies of contemporary medicine is created by an increased consumption of nicotine and growing evidence of its connection to cancer, which urges elucidation of the molecular mechanisms of oncogenic effects of inhaled nicotine. Current research indicates that nicotinergic regulation of cell survival and death is more complex than originally thought, because it involves signals emanating from both cell membrane (cm)- and mitochondrial (mt)-nicotinic acetylcholine receptors (nAChRs). In this study, we elaborated on the novel concept linking cm-nAChRs to growth promotion of lung cancer cells through cooperation with the growth factor signaling, and mt-nAChRs — to inhibition of intrinsic apoptosis through prevention of opening of mitochondrial permeability transition pore (mPTP).

**Methods:**

Experiments were performed with normal human lobar bronchial epithelial cells, the lung squamous cell carcinoma line SW900, and intact and NNK-transformed immortalized human bronchial cell line BEP2D.

**Results:**

We demonstrated that the growth-promoting effect of nicotine mediated by activation of α7 cm-nAChR synergizes mainly with that of epidermal growth factor (EGF), α3 — vascular endothelial growth factor (VEGF), α4 — insulin-like growth factor I (IGF-I) and VEGF, whereas α9 with EGF, IGF-I and VEGF. We also established the ligand-binding abilities of mt-nAChRs and demonstrated that quantity of the mt-nAChRs coupled to inhibition of mPTP opening increases upon malignant transformation.

**Conclusions:**

These results indicated that the biological sum of simultaneous activation of cm- and mt-nAChRs produces a combination of growth-promoting and anti-apoptotic signals that implement the tumor-promoting action of nicotine on lung cells. Therefore, nAChRs may be a promising molecular target to arrest lung cancer progression and re-open mitochondrial apoptotic pathways.

## Background

One of the major controversies of contemporary medicine is created by an increased consumption of nicotine and growing evidence of its connection to cancer (reviewed in [1]). Nicotine can contribute in a variety of ways to cancer survival, growth, metastasis, resistance to chemotherapy, and create a tumor-supporting microenvironment, thus implementing a "second hit" that aggravates aberrant signaling and elicits survival and expansion of cells with genomic damage [1]. The list of cancers reportedly connected to nicotine is expanding, and presently includes small- and non-small cell lung carcinomas as well as head and neck, gastric, pancreatic, gallbladder, liver, colon, breast, cervical, urinary bladder and kidney cancers ([1] and references therein).

Once limited to cigarettes, cigars, pipe tobacco and chewing or spit tobacco, nicotine-containing products today come in more flavors, forms, shapes and sizes, and with more unproven health claims. Electronic cigarettes (**eCigs**) that aerosolize nicotine without generating toxic tobacco combustion products are rapidly gaining acceptance as an alternative to conventional cigarettes with little knowledge regarding their biomedical effects [2-4]. eCig use, or vaping, allows to achieve systemic nicotine concentration similar to that produced from traditional cigarettes [5]. Although eCigs are generally recognized as a safer alternative to combusted tobacco products, there are conflicting claims about the degree to which these products warrant concern for the health of the vapers [6,7], and there is a risk of a second- and third-hand exposure to nicotine from eCigs [8]. Thus, there is an urgent need for elucidation of the molecular mechanism of oncogenic effects of inhaled nicotine to facilitate development and evaluation of safety measures for eCigs.

Nicotine can displace the autocrine and paracrine hormone-like molecule acetylcholine (**ACh**) from the nicotinic class of ACh receptors (**nAChRs**) expressed in lung cells due to its higher receptor-binding affinity. ACh is produced practically by all types of human cells, and is remarkably abundant in the lung epithelium [9,10]. Increasingly, a wider role for ACh in cell biology is being recognized, including proliferation, differentiation, apoptosis, adhesion and motility (reviewed in [11,12]). The final cellular response to ACh is determined by the delicate balance between the growth-promoting and inhibiting signals. The extracellular pool of ACh is replenished by vesicular ACh transporter secreting the ACh-containing vesicles, whereas the intracellular pool is represented mainly by free cytoplasmic ACh [13,14]. In human bronchioalveolar carcinoma cells, nicotine upregulates choline acetyltransferase and vesicular ACh transporter, thus increasing production and secretion of ACh [15]. Nicotine also can upregulate nAChR expression [16], thus shifting ACh signaling in lung cells toward the nicotinic *vs.* muscarinic physiological signaling pathways.

The nAChRs are classic representatives of superfamily of the ligand-gated ion channel pentameric receptor proteins composed of ACh binding α subunits and "structural" subunits. Lung cells can express the α1, α2, α3, α4, α5, α6, α7, α9, α10, β1, β3, β2, β4, γ, δ and ε nAChR subunits [17-22]. The differences in subunit composition determine the functional and pharmacological characteristics of the receptor pentamers formed, so that the net biological effect produced by a nicotinic agonist depends on the subtype of nAChR binding this ligand with the highest affinity. While direct involvement of α7 nAChR has been documented in the pathophysiology of lung cancer [23], α9 nAChR is known to play an important role in breast cancer [24-26]. Silencing of the expression of nAChR subunits and treatment with nAChR antagonists produce anti-tumor effects both *in vitro* and *in vivo* [15,25,27-32].

The nAChR subunit proteins can physically associate with both protein kinases and protein tyrosine phosphatases in large multimeric complexes [33]. Even a short-term exposure to nicotine activates mitogenic signaling pathways involving signaling kinases [34]. The nAChRs mediate the nicotine-dependent upregulation of genes contributing to progression of lung cancer [35-38]. Current research, however, indicates that nicotinergic regulation of cell survival and death is more complex than originally thought. The emerging picture is that a diversity of molecular signaling circuitries regulating cancer cell growth signifies cross-talk interactions between cell membrane (**cm-**)nAChRs and growth factor (**GF**) receptors (**GFRs**), and receptors to various other autocrine and paracrine mediators [1]. Additionally, modulation of functional electron transport in mitochondria has been recently found to play an important role in implementing the nicotine action interfering with chemotherapy-induced apoptosis [39].

Nicotine can permeate lung cells and activate the mitochondrial (**mt-**)nAChR subtypes found on the mitochondrial outer membrane of lung cells [40]. Activation of these receptors may inhibit opening of mPTP, which can block the initial step of intrinsic apoptosis [41-44]. The mPTP is a multi-component protein aggregate comprised by structural elements of the inner as well as outer mitochondrial membrane that form a non-specific pore permeant to any molecule of <1.5 kDa in the outer mitochondrial membrane under conditions of elevated matrix Ca^2+^. mPTP opening causes massive swelling of mitochondria, rupture of outer membrane and release of intermembrane components that induce intrinsic apoptosis, such as cytochrome c (**CytC**). Mitochondria become depolarised causing inhibition of oxidative phosphorylation and stimulation of ATP hydrolysis [45-47].

We hypothesized that the tumor-promoting activities of nicotine are implemented through two principally different mechanisms — facilitation of growth of cancer cells and prevention of their death, which results primarily from a synergistic proliferative action of cm-nAChRs with their partnering GFRs and activation of the mt-nAChRs coupled to inhibition of mPTP opening, respectively. To pin down the principal mechanisms through which nicotine contributes to lung cancer, we focused our studies of cm-nAChRs on regulation of lung cancer growth and proliferation and studies of mt-nAChRs — on cell protection from intrinsic apoptosis. We found that the growth-promoting effect of nicotine mediated by activation of α7 cm-nAChR synergizes mainly with that of epidermal GF (**EGF**), α3 — vascular endothelial GF (**VEGF**), α4 — insulin-like GF I (**IGF-I**) and VEGF and α9 — EGF, IGF-I and VEGF. We also established the ligand-binding abilities of mt-nAChRs and demonstrated that quantity of the mt-nAChRs coupled to inhibition of mPTP opening increases upon malignant transformation of lung cells. These results indicated that the biological sum of effects resulting from simultaneous activation of nAChRs on the cell membrane and mitochondria produces a combination of growth-promoting and anti-apoptotic signals that implement the tumor-promoting action of nicotine on lung cells.

## Methods

### Cells and reagents

Normal human lobar bronchial epithelial cells (**BEC**) were purchased from Life Technologies (Grand Island, NY) and the established tumorigenic line of grade IV lung squamous cell carcinoma SW900 — from American Type Culture Collection (Catalog # HTB-59; Manassas, VA). BEP2D cells — an established clonal population of HPV-18-immortalized human BEC — was a gift from Dr. Harris (NCI, NIH). For transformation, BEP2D cells were incubated for 48 h with 2 μg/ml of 4-(methylnitrosamino)-1-(3-pyridyl)-1-butanone (**NNK**; Toronto Research Chemicals, North York, ON, Canada) and then grown for 5 passages, which was sufficient to induce malignant transformation evidenced by anchorage-independent growth and tumor formation in Nu/Nu mice [48]. All types of lung cells were grown in the Cambrex bronchial cell medium without retinoic acid and used in experiments at ~80% confluence. The effects of test agents on proliferation were evaluated by directly measuring the number of viable, ie, trypan blue dye (TBD)-negative, cells using a hemocytometer. The nAChR agonist nicotine, as well as α-bungarotoxin (**αBtx**) — the specific inhibitor of the "central" subtype of the neuronal nAChRs, such as α7 [49], Mecamylamine (**Mec**) — a preferential blocker of the "ganglionic" nAChR subtypes, such as α3- and α4-made nAChRs [50], the metabolic inhibitor of ACh synthesis hemicholinium-3 (**HC-3**), which inhibits ACh synthesis by blocking cellular reuptake of its metabolic precursor choline [51], staurosporine, heat-inactivated newborn calf serum and all secondary antibodies were purchased from Sigma-Aldrich Corporation, Inc. (St. Louis, MO). Human recombinant EGF was obtained from R&D Systems, Inc. (Minneapolis, MN), IGF-I — from GenWay Biotech Inc. (San Diego, CA), and VEGF — from Abcam (Cambridge, MA). The human cytochrome c (**CytC**) immunoassay was purchased from R&D Systems and performed following the protocol provided by the manufacturer. The nicotinic radioligands (—)[N-methyl-^3^H]nicotine (specific activity 80.4 Ci/mmol), [^3^H]αBtx (specific activity 73.0 Ci/mmol) and [^3^H]epibatidine (specific activity 54.0 Ci/mmol) were purchased from GE Healthcare Bio-Sciences (Pittsburgh, PA). The antibodies to human α3, α4, α7, and α9 nAChR subunits were raised and characterized in our previous studies [52-54]. The predesigned and tested small hairpin (**sh**)RNAs targeting human CHRNA3, CHRNA4, CHRNA7 or CHRNA9, and scrambled shRNA were from OriGene Technologies (Rockville, MD).

### shRNA transfection experiments

For transfection of SW900 cells with the HuSH-29™ predesigned shRNA plasmids specific for human α3, α4, α7 and α9 nAChR subunits, we followed the standard protocol described by us in detail elsewhere [55]. Briefly, SW900 cells were seeded at a density of 1 × 10^4^ cells per well and exposed to experimental, ie, nAChR subunit gene-specific shRNA, or negative control shRNA (**shRNA-NC**) plasmids in GIBCO™ Opti-MEM I Reduced-Serum Medium (Invitrogen, Carlsbad, CA) with the TransIT®-Keratinocyte Transfection Reagent (Mirus Bio LLC, Madison, WI). The transfection was continued for additional periods of time to determine changes of the relative protein levels of each targeted nAChR subunit by immunoblotting and immunofluorescence. The maximum inhibition was achieved at 72 h after transfection (data not shown), at which point the shRNA-transfected cells were washed and exposed to test nicotine/GF combinations for 24 h. At the end of incubation, alive, ie, TBD-negative, cells were counted with a hemocytometer.

### Radioligand binding assays of mitochondrial proteins of test lung cells

The mitochondrial protein fractions were purified from large quantities of lung cell types used in this study grown in the 225 cm^2^ T-flasks employing the mitochondrial/cytosol fractionation kit from BioVision Research Products (Mountain View, CA), as described by us elsewhere [56]. Briefly, the cells were detached by a brief trypsinization, isolated by centrifugation, washed in PBS, resuspended in the Cytosol Extraction Buffer containing a mix of DTT and protease inhibitors, homogenized in an ice-cold tissue grinderm and centrifuged at 700 × *g* for 10 min at 4°C. The supernatant was re-centrifuged at 10,000 × *g* for 30 min at 4°C, and the pelleted mitochondrial fraction was resuspended in 100 μl of the Mitochondrial Extraction Buffer and used in the radioligand-binding assays following the standard protocol detailed by us elsewhere [57]. Depending on the experimental conditions (see Results), the mitochondria were exposed to either increasing concentrations of the pan-nAChR radioligand nicotine or the saturating concentrations of the preferential radioligands of the central and ganglionic nAChR subtypes, αBtx and epibatidine, respectively [58,59]. After incubation, the mitochondria were washed and solubilized with 1% SDS, the protein concentration determined by a Bradford protein assay kit (Bio-Rad Hercules, CA), and the radioactivity counted in a liquid scintillation counter. The specific binding was calculated by subtracting the non-specific binding from total binding.

### Sandwich (s)ELISA experiments

sELISA was performed as described elsewhere [60]. Briefly, ELISA plates were coated with either α3- or α7-specific rabbit antibody or non-immune rabbit IgG and blocked with 3% BSA. The lysates were applied into the coated wells for 3 h at 37°C, after which the plates were washed and incubated for additional 2 h with biotinylated anti-α3 or anti-α7 antibody (both from Antibodies-online, Inc., Atlanta, GA), followed by ExtrAvidin-Peroxidase conjugate and o- phenylenediamine dihydrochloride. The bound antibody was detected at OD 490 nm using an ELISA plate reader.

### Statistical analysis

All experiments were performed in triplicate or quadruplicate, and results expressed as mean ± SD. Statistical significance was determined using Student's *t*-test. Differences were deemed significant if the calculated *p* value was <0.05.

## Results

### The cm-nAChRs regulate proliferation of normal and malignant lung cells

**Figure 1:**
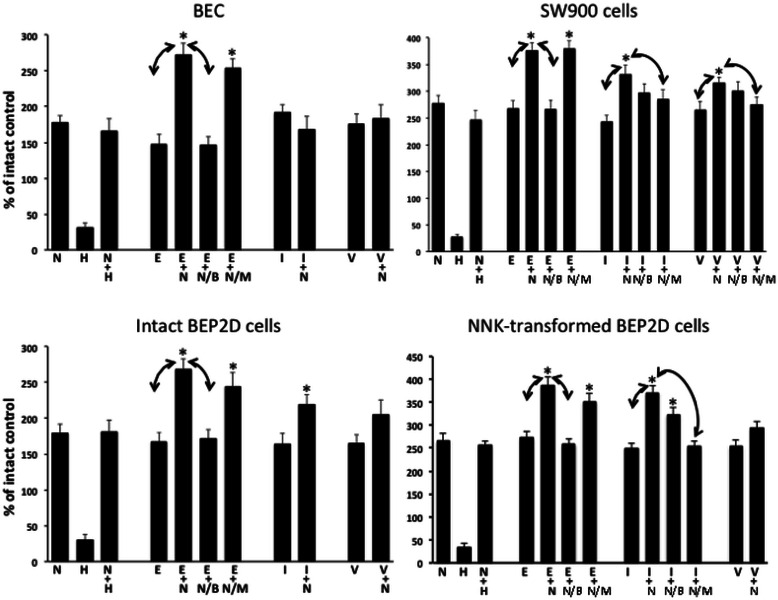
**Synergistic effects of combinations of nicotine (N) with EGF (E), IGF-I (I) or VEGF (V) on proliferation of different types of lung cells.** Alive, ie, TBD-negative, cells seeded at a density of 1 × 10^4^ per well of a 96-well plate were counted after 24 h of incubation in the absence (intact control) or presence of the optimal doses of nicotine (see text) and 10 ng/ml of each test GF. Some cells were exposed to 20 μM HC-3 (**H**) ± nicotine and some — to nicotine/GF combinations in the presence of αBtx (**B;** 1 μM) and/or Mec (**M;** 50 μM). All values significantly (p < 0.05) differed from the intact control, taken as 100%. Data are mean + SD from a triplicate sample. *Asterisk* = p < 0.05 compared to nicotine alone; *arrows* = p < 0.05 between indicated conditions.

Deprivation of cultured lung cells of auto/paracrine ACh due to treatment with HC-3 almost completely inhibited proliferation of all studied types of lung cells (Figure 1). Nicotine sustained proliferation of HC-3-treated cells (Figure 1). In a pilots study, we had determined that the effect of nicotine was cell type- and dose-dependent, with the dose of 3 μM completely restoring normal proliferation of BEC, 1 μM — intact BEP2D cells, 0.5 μM —NNK-transformed BEP2D cells and 1 μM — SW900 cells.

The ability of nicotine to restore proliferation of the HC-3-treated cells demonstrated critical role of the nicotinergic arm of cholinergic regulatory axis in implementing the growth-promoting activities of auto/paracrine ACh on lung cells.

### The growth-promoting effects of nicotine and GFs synergize

Based on the knowledge on cooperation of nicotine with GFs [1], we sought to obtain evidence that such binary systems operate in lung cells. Toward this end, we screened human GFs known to promote growth of lung cancer cells, ie, EGF [61], IGF-I [62] and VEGF [63] utilizing working concentrations of each GFs reported in the literature, and the doses of nicotine restoring proliferation of each type of lung cells under consideration (Figure 1). Rather unexpectedly, we found that different types of lung cells respond differently to combinations of nicotine with different GFs. While the proliferation rate significantly (p < 0.05) exceeding that established for each stimulant given alone was produced by a combination of nicotine with EGF in all lung cell types, the nicotine/IGF-I combination did so only in experiments with NNK-transformed BEP2D and SW900 cells, whereas the nicotine/VEGF combination — only in SW900 cells (Figure 1). When the differences between the elevated proliferation rate induced by a combination of nicotine with a particular GF significantly (p < 0.05) exceeded that induced by each stimulant given alone, we attempted to abolish the additive effect by the antagonists or predominantly α7 and non-α7 nAChRs, αBtx and Mec, respectively. Since these drugs do not penetrate the cell, they could inhibit cm-nAChRs, but not mt-nAChRs. The additive effect of nicotine to the EGF-induced proliferation could be abolished by αBtx, whereas that to the IGF-I- or VEGF-induced proliferation — by Mec (Figure 1).

These results provided the first evidence that the binary systems comprised by cm-nAChRs and GFRs facilitate growth of lung cells. The differences in the efficacies of different nicotine/GF combinations may be explained by the reputed differences in the cm-nAChR repertoires and/or their downstream signaling pathways among tested lung cell types.

### Identification of the cm-nAChR subtypes implementing synergy of nicotine with GFs

**Figure 2:**
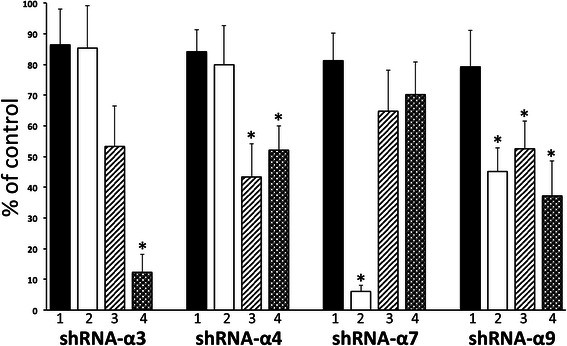
**Roles of individual cm-nAChR subtypes in implementing synergy of nicotine with GFs.** The SW900 cells transfected with either shRNA-NC (control; taken as 100%) or anti-nAChR shRNA were incubated for 24 h in the absence **(1)** or presence of combinations of 0.1 μM nicotine with 10 ng/ml of EGF **(2)**, IGF-I **(3)** or VEGF **(4)**, and then subjected to direct counting (TBD-negative cells only). Data are mean + SD from a triplicate sample. *Asterisk* = p < 0.05 compared to the untreated cells transfected with respective anti-nAChR shRNA (**1**).

To elucidate the mechanisms of cooperation of cm-nAChRs and GFRs, we focused on the cm-nAChR subtypes that might implement the synergistic growth-promoting effects of nicotine with EGF, IGF-I and VEGF in SW900 cells, because these cells were sensitive to the synergistic effects of nicotine combinations with each tested GFs (Figure 1). The involvement of a particular cm-nAChR subtype in the binary interaction with GFRs was determined based on disappearance of the additive (synergistic) effect upon functional inactivation of the cm-nAChR in question by transfection with anti-receptor shRNAs, but not shRNA-NC. In keeping with results obtained with pharmacological nAChR antagonists (Figure 1), silencing of the α7 gene selectively inhibited synergy of nicotine with EGF (Figure 2). The shRNA-α3 inhibited most effectively the nicotine synergy with VEGF, whereas shRNA-α4 — that with IGF-I and VEGF equally efficiently. Interestingly, abolishing signaling by α9 nAChRs significantly (p < 0.05) decreased the additive effect of nicotine to that of each tested GF (Figure 2).

These results indicated that α7 cm-nAChR cooperates mainly with the EGF, α3 — VEGF, α4 — IGF-I and VEGF and α9 — EGF, IGF-I and VEGF receptors.

### Differences of the ligand-binding parameters of mt-nAChRs in normal and malignant lung cells

**Figure 3:**
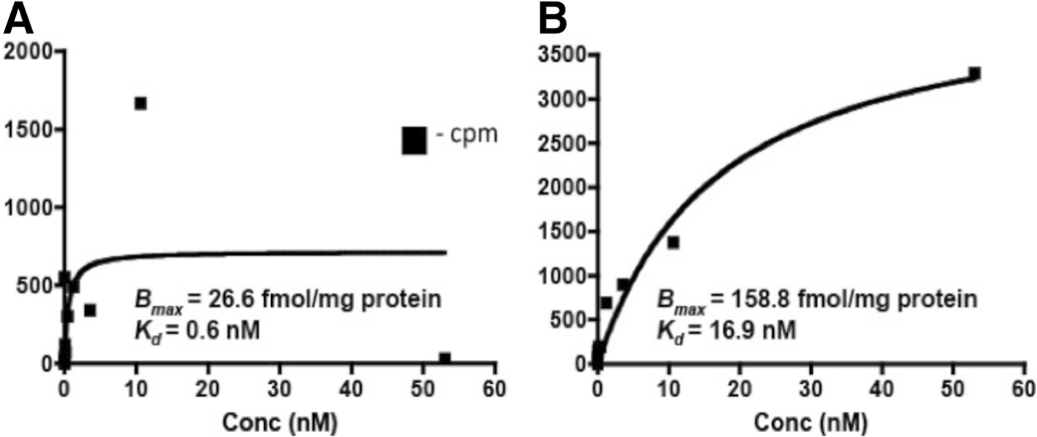
**Saturable binding of [**^**3**^**H] nicotine to mitochondria isolated from cultured BEC (A) and SW900 cells (B).** Each point represents a mean radioactivity of quadruplicate samples of purified mitochondria exposed to increasing concentrations of [^3^H] nicotine for 45 min at 0^o^ C in the absence (total binding) or presence (non-specific binding) of 1 M of non-labeled nicotine, as described in Materials and Methods.

First, we investigated whether the nAChR subunits detected on mitochondria of lung cells by sELISA [40] can form functional ligand-binding receptors, and then compared the ligand-binding parameters of mt-nAChRs in normal and malignant lung cells. Analysis of specific binding to mitochondria isolated from BEC and SW900 cells identified functional ligand-binding sites, and also demonstrated that lung cancer cells feature an increased total number of mt-nAChRs (Figure 3).

Thus, the nAChR subunit proteins expressed on the mitochondrial membrane of lung cells form functional receptors, whose number is upregulated in cancer cells.

### The oncogenic transformation alters the repertoire of mt-nAChRs in BEP2D cells

**Figure 4:**
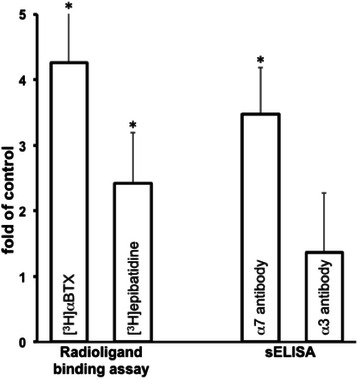
**Relative amounts ofα7 and non-α7 mt-nAChR subtypes in BEP2D cells before and after malignant transformation.** The transformation was achieved due to 48 h exposure to 2 μg/ml NNK followed by 5 passages, and confirmed in the *in vitro* and *in vivo* tumorigenicity assays (see Methods). In radioligand-binding assay, the numbers of α7 and non-α7 nAChRs were estimated by the amounts of specific binding of [^3^H] αBtx and [^3^H] epibatidine, respectively. Quadruplicate samples of mitochondria from control (intact) and NNK-transformed BEP2D cells were incubated for 30 min at 0^o^ C with saturating concentrations of the radioligands. The specific binding was calculated by subtracting the non-specific binding from total binding. In sELISA, the mitochondrial proteins were probed with anti-α7 or anti-α3 antibodies, as detailed in Methods. Results of both assays are expressed as fold of control, taken as 1. Data are mean + SD. *Asterisk* = p < 0.05 compared to respective controls.

Since malignant transformation of lung cells is associated with changes in the cm-nAChR repertoire (reviewed in [1]), we hypothesized that the repertoire of mt-nAChRs might also change. To identify possible shift in the repertoire of mt-nAChR subtypes associated with malignant transformation of lung cells, we analyzed mitochondria from intact *vs.* NNK-transformed BEP2D cells using a combination of radioligand-binging assay and sELISA. By the former technique, we determined specific binding of the preferred radioligands of α7 and non-α7 nAChRs, [^3^H] αBtx and [^3^H] epibatidine, respectively. By the latter technique, we quantitated relative amounts of α7- and α3-made nAChRs employing our α7- and α3-selective antibodies. The radioligand-binging assay demonstrated that mitochondria from the NNK-transformed BEP2D cells featured increased amounts of both α7 and non-α7 receptors, as judged from a 4.3-fold increase of the [^3^H] αBtx- and a 2.4-fold increase of the [^3^H] epibatidine-binding sites, compared to control, non-transformed BEP2D cells (Figure 4). By sELISA, the relative amounts of α7 and α3 subunit proteins in the NNK-transformed BEP2D cells increased by 3.5- and 1.4-fold, respectively (Figure 4).

Thus, we obtained direct evidence that malignant transformation of lung cells is associated with an increased expression of α7 mt-nAChR and, perhaps, some other mt-nAChR subtypes that may be coupled to inhibition of mPTP opening [40].

### Identification of the lung mt-nAChR subtypes coupled to inhibition of apoptosis

**Figure 5:**
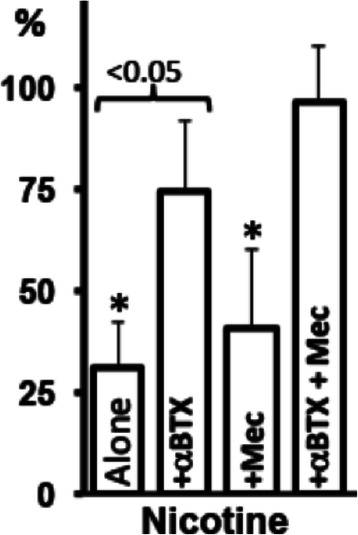
**Nicotinergic effects on CytC release from BEC mitochondria.** The mitochondria freshly isolated from BEC were exposed to 1 μM staurosporine (control) and incubated in triplicate for 45 min at 30 ° C with 10 μM nicotine in the absence or presence of αBtx (1 μM) and/or Mec (50 μM). The mitochondria were pelleted and the CytC concentration was measured in the supernatants, as described in Materials and Methods. Data are mean + SD. *Asterisk* = p < 0.05 compared to treatment with staurosporine alone, taken as 100%.

To obtain an insight into the roles of different mt-nAChR subtypes in the inhibition of mPTP opening in lung cells, we measured effects of nicotinic ligands on the staurosporine-induced CytC release, which is known to be associated with mPTP opening and activation of the intrinsic apoptotic pathway [64]. In keeping with published reports [40,41,65], we observed that nicotine significantly (p < 0.05) inhibited staurosporine-induced CytC release from naked mitochondria isolated from BEC (Figure 5). The ability of nicotine to block CytC release was significantly (p < 0.05) and insignificantly (p > 0.05) diminished in the presence of αBtx and Mec, respectively. The mixture of αBtx and Mec completely abolished the anti-apoptotic effect of nicotine (Figure 5).

These results demonstrated that activation of lung mt-nAChRs by nicotine inhibits apoptogen-induced mPTP opening, and indicated that both α7 and non-α7 mt-nAChR subtypes may be involved in the anti-apoptotic action of nicotine.

## Discussion

This study elaborated on the novel concept linking cm-nAChRs to growth promotion of lung cancer cells through modification of GF signaling, and mt-nAChRs — to inhibition of apoptosis due to prevention of mPTP opening. The obtained results provided new insights into the molecular mechanisms of nicotinergic regulation of normal and malignant lung cells. We demonstrated for the first time that the nAChR-mediated growth-promoting effects of nicotine synergize with those of EGF, IGF-I and VEGF. The causative role of activation of cm-nAChRs in the growth-promoting action of nicotine was illustrated by the ability to abolish its effect using the cell membrane-impermeable nAChR antagonists. Different cm-nAChR subtypes implemented the synergistic action of nicotine with GFs in different types of lung cells. Also for the first time, we demonstrated that mt-nAChRs implement the anti-apoptotic activity of nicotine that permeates lung cells. Thus, it appears that the oncogenic action of nicotine harbors both the growth-promoting and anti-apoptotic signals emanating from the cell membrane and the mitochondrial membrane due to activation of cm-nAChRs and mt-nAChRs, respectively.

Concerns about safety of nicotine-containing products necessitates research of the molecular mechanisms of nicotine action on the tissues prone to develop tobacco-related malignancy, such as lungs. The additive oncogenic effect of nicotine is best illustrated in the lung cancer model in A/J mice, wherein nicotine increases both the numbers and the size of tobacco nitrosamine-initiated lung tumors, and decreases survival probability [23,34,66]. Furthermore, while smoking is an independent predictive factor of chemoresistance of lung cancer [67], silencing of nAChRs in the non-small-cell lung carcinoma cell lines suppresses nicotine-dependent chemoresistance [68]. Therefore, it is currently believed that nAChRs may be a novel drug target for prevention and treatment of cancers [69-73].

Although nAChR is an ion channel mediating influx of Na^+^ and Ca^2+^ and efflux of K^+^, its activation by a ligand, such as nicotine, elicits both ionic and non-ionic signaling events regulating phosphorylation and dephosphorylation of target proteins. Altogether, the downstream signaling from cm-nAChRs has been shown to activate protein kinase C isoforms, Ca^2+^/calmodulin-dependent protein-kinase II, Jak2, phosphatidylinositol-3-kinase, JNK, phospholipase C, EGFR kinase, Rac, Rho, p38 and p44/42 MAPK, as well as the Ras-Raf1-MEK-ERK pathway [74-87]. Notably, stimulation of α7 cm-nAChR in keratinocytes triggers two complementary pathways. The Ras-Raf1-MEK1-ERK cascade culminates in up-regulated expression of the gene encoding STAT3, whereas recruitment and activation of the tyrosine kinase JAK2 phosphorylates it. Thus, cm-nAChRs couple several non-receptor kinases that can activate different signaling cascades merging with GFR pathways, with the signal flow ending at the level of specific transcription factors. For instance, it is well-documented that nicotine accelerates wound healing by synergizing with and mimicking the effects of various GFs [88-90]. Nicotine can also upregulate expression of fibroblast growth factor (**FGF**)1, FGF1 receptor, FGF2 and VEGF [38,83,91-95]. Accordingly, nAChR inhibition reduces FGF2 and VEGF upregulation [73,96]. In turn, FGF2 and IGF-I alter the cm-nAChR expression level and clustering [97,98], which can modify biological effects of auto/paracrine ACh, and nicotine.

It has been well-documented that nAChRs can mediate the nicotine-dependent upregulation of proliferative and survival genes, thus contributing to the growth and progression of lung cancer cells *in vitro* and *in vivo* [35-37]. In the present study, we demonstrated that a combination of nicotine with EGF, IFG-I or VEGF increases lung cell proliferation above the levels established for each stimulant given alone. Since nicotine can exert its biological effects due to binding to the cm-nAChRs functionally linked to GFRs (reviewed in [1]), its tumor-promoting activities may, therefore, rely on the synergy of the cm-nAChR- and GFR-coupled signaling events. The homomeric α7 nAChRs, homo- and/or heteromeric α9-containing nAChRs as well as the α3- and α4-made nAChR subtypes, all appeared to be involved in the binary circuitries with GFRs facilitating lung cancer cell growth. Thus, it has become apparent that activation of cm-nAChRs primarily triggers signaling events accelerating tumor growth, whereas activation of mt-nAChRs primarily protects tumor cells from apoptosis. Admittedly, such "assignment" is somewhat artificial, since cm-nAChRs can also inhibit apoptosis by upregulating anti-apoptotic factors (eg, [99]).

Although nicotine can freely permeate epithelial cells and elicit pathobiological effects via intracellular mechanisms [100-104], up until recently the pro-survival activities of nicotine had been attributed exclusively to activation of cm-nAChRs. However, It has been recently demonstrated that nAChR subunits are also expressed on the mitochondrial outer membrane [42,43]. The nAChR-subunit antibodies visualized the α3, α4, α7, β2 and β4 subunits forming in the mitochondria of lung cells the nAChRs that non-covalently connect to voltage-dependent anion channels and control CytC release by inhibiting mPTP opening [40,41]. We have chosen staurosporine as an apoptogen, because it increases mitochondrial membrane potential and induces mitochondrial swelling and CytC release, which can be blocked by an inhibitor mPTP opening [64,105]. Demonstration of the mt-nAChRs preventing mPTP opening was in keeping with independent reports about both the presence of nAChRs on mitochondria [106] and the mitochondria-protecting effects of nicotine [107,108].

Changes of the mt-nAChR expression patterns associated with malignant transformation of lung cells may play an important role in the biology of cancer cells. We demonstrated that mitochondria of the malignant lung cells SW900 expressed more nAChRs than normal BEC, which is in keeping with the notion that cancer progression is associated with overexpression of nAChRs (reviewed in [69,71,109]). An increase of mt-nAChR numbers may allow malignant cells to bind a higher than normal amounts of auto/paracrine ACh or nicotine. In the cytosol, nicotine can shift the dynamic equilibrium of the physiological regulation of cell survival and death, because it is insensitive to the regulatory action of intracellular acetylcholinesterase that hydrolyzes ACh in the cytoplasm [110] and thus exerts the physiological control of anti-apoptotic action of mt-nAChRs, similar to the effect of cell membrane-anchored acetylcholinesterase hydrolyzing extracellular ACh.

It has been documented that a switch of the predominant nAChR expression pattern occurs during malignant transformation of the cells (reviewed in [70,71]), indicating that the effects of auto/paracrine ACh on cancer cells might differ from its effects on non-malignant cells, even if they are situated next to each other in the same tissue. The same holds true for nicotine, which has a higher nAChR-binding affinity than ACh. The cumulative results of our radioligand-binding assay and sELISA indicated that malignant transformation of lung cells was associated with an upregulated expression of predominantly the α7 mt-nAChR subtype. Notably, the degree of increase of mt-nAChRs detected by a radioligand was higher than that detected by a corresponding antibody. This can be explained by the fact that, in contrast to the nAChR subunit-selective antibody, each radioligand can label more than one nAChR subtype. Since the specificity of many antibodies against nAChRs was put in doubt [111,112], we had verified specificity of our antibodies in the α7 and α3 knockout mice (data not shown).

## Conclusions

The results of our experiments showing cooperation between the binary signaling networks of specific cm-nAChRs and GFRs, on the one hand, and the data on inhibition of mPTP opening by mt-nAChRs on the other, indicate that the biological sum of simultaneous activation of nAChRs on the cell membrane and the mitochondrial membrane by nicotine produces combined growth-promoting and anti-apoptotic effects. Noteworthily, inhibition of nAChR expression has been shown to attenuate nicotine- or tobacco nitrosamine-induced cell proliferation *in vitro* and/or *in vivo* (reviewed in [1,109]). Therefore, elucidation of this novel mechanism of tumor promoting action of nicotine should pinpoint the lung cm-nAChR and mt-nAChR subtypes that may become a promising molecular target to prevent, reverse, or retard lung cancer progression by receptor inhibitors. Since nicotine can protect cancer cells from apoptosis and elicit chemoresistance (reviewed in [1]), learning the pharmacology of nicotinergic regulation of mPTP opening should allow to re-open the mitochondrial apoptotic pathways, thus restoring sensitivity of lung cancer to chemotherapy.

## Abbreviations

ACh: Acetylcholine
BEC: Bronchial epithelial cells
αBtx: α-bungarotoxin
cm-nAChR: Cell membrane nAChR
CytC: Cytochrome c
eCig: Electronic cigarette
EGF: Epidermal growth factor
GF: Growth factor
GFR: Growth factor receptor
IGF-I: Insulin-like growth factor
Mec: Mecamylamine
mt-nAChR: Mitochondrial nAChR
nAChR: Nicotinic ACh receptor
NNK: 4-(methylnitrosamino)-1-(3-pyridyl)-1-butanone
sELISA: Sandwich ELISA
shRNA: Small hairpin RNA
TBD: Trypan blue dye
VEGF: Vascular endothelial growth factor
shRNA-NC: Negative control shRNA
